# Validation of Scolioscan Air-Portable Radiation-Free Three-Dimensional Ultrasound Imaging Assessment System for Scoliosis

**DOI:** 10.3390/s21082858

**Published:** 2021-04-19

**Authors:** Kelly Ka-Lee Lai, Timothy Tin-Yan Lee, Michael Ka-Shing Lee, Joseph Chi-Ho Hui, Yong-Ping Zheng

**Affiliations:** Department of Biomedical Engineering, The Hong Kong Polytechnic University, Hong Kong; kelly.lai@polyu.edu.hk (K.K.-L.L.); timothy.lee@connect.polyu.hk (T.T.-Y.L.); i-ka-shing.lee@polyu.edu.hk (M.K.-S.L.); joseph-ch.hui@polyu.edu.hk (J.C.-H.H.)

**Keywords:** scoliosis, 3D ultrasound, ultrasound imaging, portable 3D ultrasound, radiation-free

## Abstract

To diagnose scoliosis, the standing radiograph with Cobb’s method is the gold standard for clinical practice. Recently, three-dimensional (3D) ultrasound imaging, which is radiation-free and inexpensive, has been demonstrated to be reliable for the assessment of scoliosis and validated by several groups. A portable 3D ultrasound system for scoliosis assessment is very much demanded, as it can further extend its potential applications for scoliosis screening, diagnosis, monitoring, treatment outcome measurement, and progress prediction. The aim of this study was to investigate the reliability of a newly developed portable 3D ultrasound imaging system, Scolioscan Air, for scoliosis assessment using coronal images it generated. The system was comprised of a handheld probe and tablet PC linking with a USB cable, and the probe further included a palm-sized ultrasound module together with a low-profile optical spatial sensor. A plastic phantom with three different angle structures built-in was used to evaluate the accuracy of measurement by positioning in 10 different orientations. Then, 19 volunteers with scoliosis (13F and 6M; Age: 13.6 ± 3.2 years) with different severity of scoliosis were assessed. Each subject underwent scanning by a commercially available 3D ultrasound imaging system, Scolioscan, and the portable 3D ultrasound imaging system, with the same posture on the same date. The spinal process angles (SPA) were measured in the coronal images formed by both systems and compared with each other. The angle phantom measurement showed the measured angles well agreed with the designed values, 59.7 ± 2.9 vs. 60 degrees, 40.8 ± 1.9 vs. 40 degrees, and 20.9 ± 2.1 vs. 20 degrees. For the subject tests, results demonstrated that there was a very good agreement between the angles obtained by the two systems, with a strong correlation (R^2^ = 0.78) for the 29 curves measured. The absolute difference between the two data sets was 2.9 ± 1.8 degrees. In addition, there was a small mean difference of 1.2 degrees, and the differences were symmetrically distributed around the mean difference according to the Bland–Altman test. Scolioscan Air was sufficiently comparable to Scolioscan in scoliosis assessment, overcoming the space limitation of Scolioscan and thus providing wider applications. Further studies involving a larger number of subjects are worthwhile to demonstrate its potential clinical values for the management of scoliosis.

## 1. Introduction

Scoliosis is three-dimensional (3D) spine deformity including structural, lateral, rotated curvature [1]. Idiopathic scoliosis is the case with scoliosis that develops in childhood spontaneously, and the majority of idiopathic scoliosis occurs from age 10 to 16, which is known as adolescent idiopathic scoliosis (AIS) [2]. The prevalence of AIS is 0.47%–5.2% of the general population and varies by regions, and the prevalence among girls is twice higher than that of boys [3]. In Hong Kong, the prevalence of AIS is 3%–4% [4,5] and that in China is about 5% [6]. Once the spine deformity developed, it may or may not progress. The progressive rate may be high that up to 10 degrees per year [7]. Risk factors include age, bone maturity, sagittal profile, and scoliosis apex location [8,9]. Girls suffer from a higher risk of progression [10]. Progressed scoliosis affects the appearance of adolescents and the imbalance often causes back pain. Severe scoliosis causes compression onto nerves, heart or lungs, leading to heart and lung problems [11,12,13]. A recent study demonstrated a group of untreated patients with AIS longitudinally and only 29.1% of them showed curve progression [14]. It also reported that only 17% of diagnosed patients with AIS have curve progression that require medical intervention [14]. To find out those progressed cases for applying medical treatment, frequent monitoring for detecting spine curvature progression is needed for AIS patients until skeletal maturity [15,16].

Quantitative assessment of curve severity in scoliosis is important for confirmation of diagnosis, management planning, prognostication, and monitoring of disease progression and treatment outcomes [17]. Radiograph-based Cobb’s method is regarded as the gold standard for assessing the severity of AIS. AIS patients normally have to undergo regular X-ray assessment every four to six months until skeletal maturity is reached. Cobb angle measurement in the frontal plane using standing postero-anterior X-ray radiograph is the gold standard for scoliosis evaluation [17,18]. A change of not less than five degrees in Cobb angle is an indicator of curve progression despite inter- and intra-observer variability on measuring the curves in radiographs [19]. Though the X-ray radiograph is the gold standard for scoliosis evaluation, it poses radiation risks. Previous studies reported that radiation over repeated exposures to radiographic assessment may increase the risk of breast cancer in girls with scoliosis [20,21,22]. In addition, radiographic diagnostics in childhood contributes significantly to leukemia and prostate cancer [23]. Treatment and management decisions for scoliosis are usually made with consideration between radiation exposure dose and disease monitoring frequency [24]. Therefore, regular check-up of scoliosis is suggested to at least six months intervals though the curve progression is fast [25]. With the radiation hazards, the patients could not undergo radiographic assessment frequently, making it difficult to perform close monitoring for the disease progression and treatment outcomes.

There is a low-dose biplanar X-ray imaging system, EOS, available in recent years. It uses slot-scanning technology to produce high-quality images with less radiation than conventional imaging techniques [26]. Previous studies reported the effectiveness and efficiency of this technology and found that it reduced the radiation dose required to obtain a 2D image of the spine by 8 to 10 times with no significant difference in diagnostic information when comparing with traditional X-ray [27,28]. The radiation dosage of the EOS is considerably lower than traditional radiography, but it is still not negligible, and thus the EOS system needs to be installed in a special X-ray shielding room. Using the EOS system for scoliosis longitudinal follow-up, there is still an accumulation of radiation dose that could lead to adverse effects related to diagnostic radiation. Moreover, the costs of the machine and renovation of the room is high, thus affecting its wide adoption.

There are also several radiation-free technologies available but not commonly used clinically due to various limitations of the technologies. Surface topography is a non-invasive method to investigate the 3D shape of the torso back surface. The abnormal torso shape usually correlates with scoliosis and this assumption is used when using this method for scoliosis diagnosis. Studies reported the attempt of using surface topography to estimate the Cobb angle and to monitor its changes with various levels of success [29,30,31]. However, the actual internal anatomical structures vary among individuals and the internal alignment of spine cannot be directly assessed, thus the assessment of scoliosis is not accurate enough [29,32,33]. DIERS formetric 4-dimensional (4D; DIERS Medical Systems, Chicago, IL, USA) is one of the surface topography systems developed in recent years and commercially available, and this technique is also named rasterstereography. Several studies reported its applications for 3D spinal measurements including coronal curvatures, sagittal curvatures, and vertebral rotation [33,34]. A recent study also reported the reliability of using DIERS for reconstruction of spinal deformities in patients with severe AIS [35]. Since the reconstruction technology is based on the assumption that surface profile of spine reflects the internal anatomical structure of spine, the obtained spinal profile cannot be very accurate [36]. A recent study with 192 participants reported that the DIERS machine demonstrated moderate accuracy in measuring the scoliotic deformity and low accuracy in monitoring the curve progression, thus suggesting to use DIERS only for early screening in large adolescent populations [37]. MRI technology can be used for the 3D assessment of spine curvature and it is a radiation-free technology. It can provide spine anatomical information together with muscular information and neurological information [38,39,40]. The MRI technology has been used for evaluation of 3D spine deformities including vertebrae segmental deformity and neuraxial abnormalities. However, traditional MRI scanning procedure requires patients to be assessed in supine postures while standing magnetic resonance imaging requires specific installation space and long operating time, and it is now widely accessible yet [41].

Ultrasound imaging is an inexpensive, radiation-free and highly portable modality allowing spine monitoring at places without conventional medical imaging devices, thus making ultrasound systems more accessible and affordable for patients when compared with MRI or radiography [41]. The working principle of ultrasound imaging is that ultrasound is reflected by the cortical surface of bones, thus providing clear images and topographic information [42]. Several free-hand 3D ultrasound imaging systems for spine assessment have been reported in the literature [41,43,44,45,46,47,48,49,50,51]. Ultrasound systems can be installed in spaces with the least limitation and be operated at low cost [52]. One system has become commercially available, specifically for assessing spinal curvatures, known as Scolioscan (Model SCN801, Telefield Medical Imaging Ltd., Hong Kong). This 3D ultrasound imaging system is composed of an ultrasound scanner with a specific linear probe, a designated frame structure, an electromagnetic spatial sensing device and the dedicated software. The 3D ultrasound imaging of the spine is achieved through freehand scanning of the ultrasound probe with electromagnetic spatial sensing device for detecting the position and orientation of the probe. During scanning, the ultrasound probe is moved from bottom to top of the back covering the whole spine area, and B-mode images are collected with corresponding position and orientation information. The data recorded are used for 3D image reconstruction and forming coronal view images of the spine using volume projection image (VPI) method [49]. The VPI method is to obtain an averaged intensity of all voxels of the volumetric image within a selected depth of approximately 10 mm along the antero-posterior direction to form an image in the coronal plane, with non-planar re-slicing technique [53]. The Scolioscan system has been widely investigated for its potentials for the measurement of scoliotic curvatures [47,48,49,53,54,55,56], forward bending study [57], classification of structural and non-structural curve [58], and screening for scoliosis [59]. The results of these studies showed that intra- and inter-rater reliability of the 3D ultrasound system was good enough and comparable to the results obtained from traditional radiographs. However, available Scolioscan and other reported 3D ultrasound imaging systems for scoliosis are comparatively large devices with some space needs, thus cannot be widely installed in clinics with small space, such as in Hong Kong. Moreover, these reported systems are not portable and need specific installation procedures. There is a compact and wireless freehand 3D ultrasound real-time spine imaging system reported with a pilot test recently [60].

**Figure 1:**
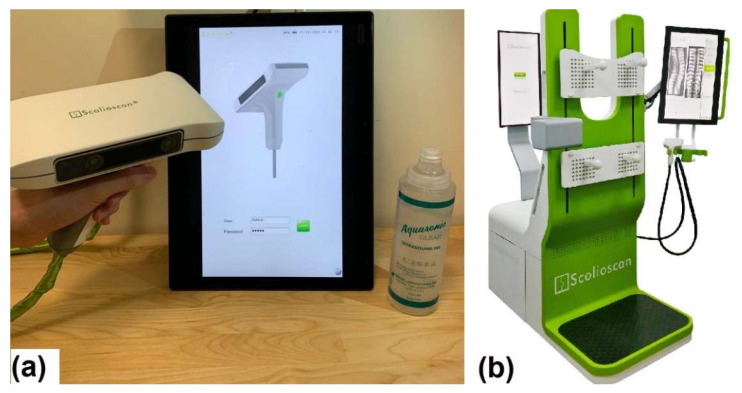
Pictures showing (**a**) portable 3D ultrasound imaging system Scolioscan Air and (**b**) conventional 3D ultrasound imaging system Scolioscan [53].

The aim of this study is to validate a newly developed portable 3D ultrasound imaging system for scoliosis assessment, which adopted the latest palm-sized ultrasound imaging module as well as a finger-sized optical 3D tracking device. This allows us to make the entire system hand-held and working together with a tablet PC. With its portable feature, assessment using Scolioscan Air can save patients’ traveling costs and time, as well as the installation and operation space. Since the device is so small (probe size of 182 × 92 × 150 mm and net weight of 450 g), we named it as Scolioscan Air. We conducted a validation study for Scolioscan Air to verify whether it could provide coronal images satisfied for scoliosis assessment as it was designed. The measurement of scoliotic curvature for the obtained coronal images was conducted and compared against the commercially available 3D ultrasound imaging system for scoliosis assessment, Scolioscan [53] (Figure 1). The results would help Scolioscan Air to be launched as a medical device, together with other required tests, including safety test, biocompatibility test, electromagnetic emission test, etc. [61].

## 2. Materials and Methods

### 2.1. Scolioscan Air System

**Figure 2:**
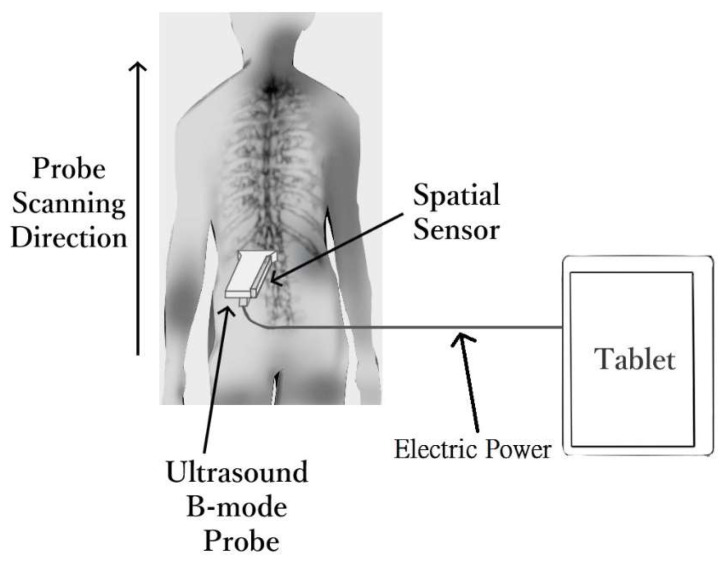
Technical diagram for the portable 3D ultrasound imaging system Scolioscan Air.

This portable 3D ultrasound imaging system was comprised of a scanning probe, which further included a case with handle, a palm-sized linear ultrasound module with a transducer of 75 mm in width and central-frequency of 7.5 MHz (UW-1C, Sonoptech, Beijing, China), a Realsense depth tracking camera (T265, Intel, Santa Clara, CA, USA) to obtain the 3D spatial data of the probe, and a tablet PC installed with a dedicated program for imaging and data collection, processing, 3D reconstruction, visualization, and curvature measurement (developed in C++). The depth camera was mounted onto one side of the ultrasound probe, with both connected to the tablet PC with a USB cable (Figure 2). When the USB cable was plugged into the tablet PC, the ultrasound module and the tracking camera are powered up and provided B-mode images and the 3D spatial information of the probe, respectively. B-mode refers to an ultrasound imaging mode that provides two-dimensional ultrasound image with the brightness of each pixel indicating the amplitude of the ultrasound echo signal received by the ultrasound transducer. Diagnostic ultrasound in B-mode allows visualization and quantification of anatomical structures [62]. The tracking camera consisted of two main components: fisheye cameras and inertial measurement units. It provided the localization and mapping solution and gave the 3D data. The sensors for spatial information detection have been optimized and calibrated in factory. During operation, the tracking camera processes the vision and motion to undergo the visual simultaneous localization and mapping (vSLAM) algorithm with the self-contained chips. The vSLAM algorithm is vision- and odometry-based, which enables low-cost navigation in cluttered environments. vSLAM can handle dynamic changes in the environment, with initial mapping. It detects the lighting changes, moving objects and/or people, and recovers quickly from different sources of disturbances [63]. It outputs the 3D spatial data to tell the position and the orientation of the tracking camera. The tracking camera undergoes self-checking to check the hardware connection once it was switched on. By collecting environmental data and running its internal V-SLAM algorithms, a value of confident level 0, 1, 2, or 3 is returned. Sufficient visual feature points have to be obtained otherwise vSLAM would estimate low confidence spatial information. In the Scolioscan Air system, a green signal would be shown on the user interface of the software when the tracking camera functioning with a high confident level (value 3), which is calculated by the sensor based on the reference map. The Realsense tracking camera uses its embedded processor to build an optical 3D map of the environment for spatial tracking purposes. Before it can perform accurate tracking, the tracking camera should first be moved along the intended tracking path for several routes to build the reference map. Operators were required to check out the status of the tracking camera with smooth tracking path before any examination began. In addition, the tracking camera works better in non-plain and non-repeating textured environment, and avoids targeting moving objects during trackings, such as a moving fan or a moving body in its viewing range, as it would affect the tracking accuracy. The origin of the 3D coordinate system would be reset when the operator clicked the “Start” button in the software user interface, and the probe should be placed at the starting point of the scan. Calibration of the offset from the spatial sensor to the ultrasound image would only be needed once for each Scolioscan Air device, which was done before the probe was used in this study. Other than the tracking camera, the other operation procedure of Scolioscan Air is almost the same as the Scolioscan system. For Scolioscan, Cheung et al. [49] introduced the 3D image reconstruction method and the formation of coronal imaging using volume projection imaging (VPI), and Zheng et al. [53] has introduced the basic operation and user interface of its software.

### 2.2. Tests for Angle Phantom

**Figure 3:**
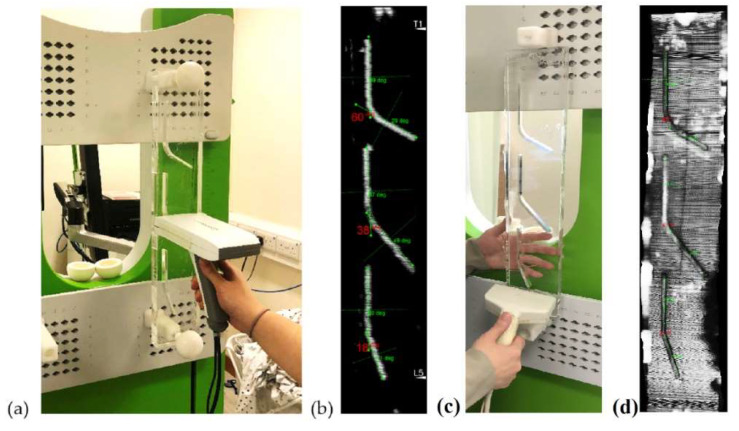
(**a**) A typical setup for scanning the plastic angle phantom using Scolioscan Air; (**b**) A typical image obtained from the angle phantom showing the three angles (60, 40, 20 degrees by design) and lines drawn for the angle measurement manually; (**c**) Same setup for scanning the phantom using Scolioscan; (**d**) A typical image obtained using Scolioscan with the same scanning and measurement procedures.

A plastic bar with three-angle structures built-in was used to evaluate the accuracy of measurement (Figure 3a). The three angles were designed as 60, 40, and 20 degrees, which are critical for scoliosis monitoring. Corrective brace treatment is considered by orthopedic doctors when the scoliotic curve reaches 20 degrees, together with the consideration of the patient’s bone maturity and lifestyle [64]. Surgical treatment may be considered to correct the scoliotic curve when it reaches 40, together with other criteria, such as bone maturity [64]. Pulmonary function abnormalities are usually detectable when the scoliotic curve is reaching 60 degrees [65]. When the probe was scanned over the phantom angle with the ultrasound beam perpendicularly to the plastic surface, ultrasound echoes would form the three angle profiles in the projection ultrasound images (Figure 3b). Then the angles were manually measured. To demonstrate that the probe could be used for scanning in different orientations, the angle phantom was placed along 10 different orientations in 3D space, with the tracking camera facing different backgrounds in the room. For each orientation, three scans were conducted. The same setup for scanning the plastic angle phantom and angle measurement procedures were carried out using the conventional Scolioscan system (Figure 3c,d). The image contrasts in Figure 3b,d were different due to different settings used during image acquisition, but this would not affect the angle measurement.

### 2.3. Subjects

Briefly, 19 volunteers with different severities of scoliosis were recruited for this study. Ethical approval for human subject study was obtained from the author’s institution before implementation of the test. All subjects (and their guardians for those below 18 years old) were given full explanation and written informed consent was obtained prior to participation in the study. Patients with BMI higher than 25.0 kg/m^2^ were excluded as high body mass index (BMI) might lead to poor ultrasound image quality for the 7.5 MHz probe used in this study. Patients with surgery done on spine and patients with spine fracture or wound that affect the application of the probe of the 3D ultrasound system during scanning were excluded as these conditions might affect ultrasound scanning. Subjects who could not stand steadily on the assessment area during the examination or subjects with allergy to the aqueous gel used for ultrasound scanning were also excluded.

### 2.4. Preparation for Subject

**Figure 4:**
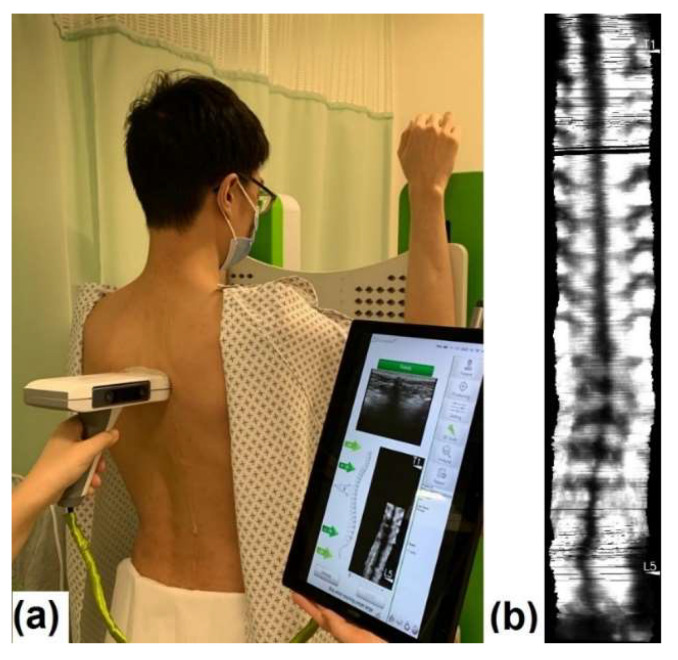
Assessment by portable 3D ultrasound imaging system Scolioscan Air: (**a**) subject being scanned and software interface shown during scanning and (**b**) typical resulting images of a scoliosis patient.

Subjects were requested to undress upper garments and shoes before the scanning session for the ease of scanning, and would wear a customized garment with their back shown for scanning. All metallic wears, electronics goods, magnets, and any possible ferromagnetic materials on subjects were removed, as the conventional Scolioscan system used for comparison used an electromagnetic tracking sensor that could be interfered by these materials. This procedure is not necessary for Scolioscan Air scanning. For the scanning with Scolioscan Air, subjects are normally asked to stand on floor with a horizontal surface facing a vertical wall or supporting surface, with their upper arm against the wall keeping elbow joint 90 degrees for body support during scanning. In this study, subjects were asked to use the supporting plate provided by the Scolioscan machine as the wall to support them (Figure 4a), and this was used for the scanning by both systems. Subjects were required to maintain his/her posture stable and their eye level horizontally throughout the scanning process.

### 2.5. Subject Examination

Ultrasound aqueous gel was used for acoustic coupling between the probe and skin. A gel warmer was used for warming the aqueous gel to keep it near body temperature. The operator applied aqueous gel to fill the spinal furrow and cover all the extent where the probe would sweep. The tracking camera and the ultrasound module were activated after the gel application. Prior to scanning, self-checking for the system was performed. Gain and dynamic range settings of the ultrasound machine were adjusted by the operator by viewing the B-mode images at the positions around T1, T12, and L5 to obtain the best overall B-mode images. The scanning depth was set at 6.0 cm in this study. After adjusting the ultrasound scanner setting, the probe was located at a level below L5 spinous process as the initiation scanning point and the operator would steer the probe up from L5 to a location slightly beyond T1 spinous process to complete the scanning process, with an average scanning speed of 1–2 cm per second. Operators should keep a comparatively constant scanning speed of 1–2 cm/s^−1^ by checking the real-time display of the speed indicator in the user interface of the software. In addition, more ultrasound coupling gel could be used to ensure smooth scanning. During the scanning, the probe was manually controlled by the operator to be as perpendicular as possible to the skin surface and following the profile of spine. The operator continuously monitored the skin surface topography and the probe orientation so as to keep them nearly perpendicular to each other. A preview of coronal image of the spine was provided in real-time during scanning. The subjects were instructed to keep relaxed and stable during the examination. The B-mode images and their corresponding spatial position and orientation data captured were then be saved and reconstructed as coronal ultrasound images using the volume projection approach for further evaluation of the spine curvature and shown on the software interface for further measurement (Figure 4b).

After the assessment by the portable 3D ultrasound imaging system, subjects were arranged to undergo the scanning using the conventional Scolioscan system with the same standing posture as the Scolioscan Air scanning. After the data were obtained, the subject and the ultrasound probe would be cleaned with tissue paper for removing the gel, the subject would be arranged to dress.

### 2.6. Data Processing

The captured sequential B-mode images with corresponding spatial position and orientation data were saved into a single file. VPI method was used and non-planar re-slicing technique was applied when forming the coronal images from the 3D spine volume [49]. The position of a non-planar VPI plane was defined according to a preset distance profile with the skin surface as a reference in each B-mode image. The volume projection image in the coronal plane would be finally formed and could be used to reveal the spine features at different depths measured from the patient skin surface. With the customized VPI method, nine images of various depths following the skin surface curve profile were produced [49]. Then, the best layer showing the most bony features was manually selected. A recent study reported the potential of using automatic selection of the best layer with encouraging results [66].

### 2.7. Angle Measurements and Study Design

**Figure 5:**
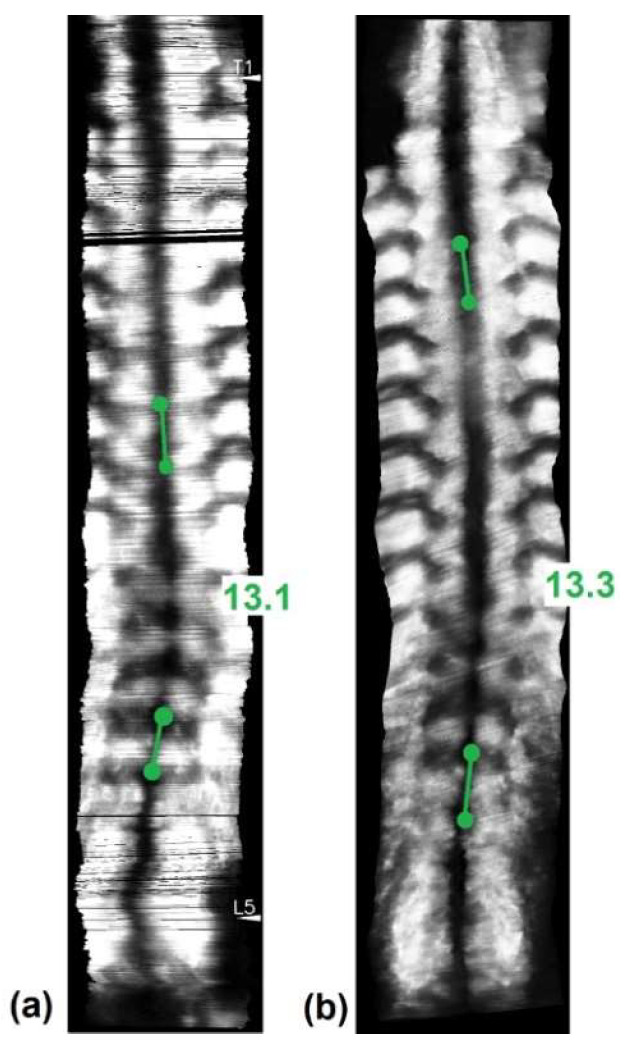
Coronal ultrasound images formed using the skin surface curve VPI method with SPA measurement on (**a**) Scolioscan Air and (**b**) Scolioscan.

Each coronal image formed by the 3D ultrasound contained a spinal profile formed by spinal process ultrasound shadow, which could be found near the mid-line of the image. The two most turning portions of a scoliotic curve were identified in each image as the most tilted vertebrae for curvature measurement. Lines were drawn along the dark mid-line at the upper most tilted vertebra and the lower most tilted vertebra of a selected curve. The angle measured with these two lines was defined to be the ultrasound spinal process angles (SPA). The software of Scolioscan and Scolioscan Air both provided the measurement functions. The same measurement protocol was carried out on images obtained by both systems (Figure 5). A study has demonstrated that the SPA measurement protocol on 3D ultrasound coronal images has very good intra- and inter-rater reliability with an intra-class correlation coefficient (ICC) larger than 0.94 and 0.88, and very good intra- and inter-operator reliability with ICC larger than 0.87 respectively [53]. The SPA results obtained by the two systems were compared and analysed.

### 2.8. Statistical Analysis

For the angle phantom test, the average and standard deviation of angles were obtained based on the 10 × 3 sets (10 different postures, each with three repeated tests) of measurement data. Linear correlation was conducted between the ultrasound SPA obtained from Scolioscan Air and Scolioscan for the 19 patients. A correlation coefficient of 0.25 to 0.50 indicates poor correlation, 0.50 to 0.75 indicates moderate to good correlation, and 0.75 to 1.00 indicates very good to excellent correlation [67]. Bland–Altman plots were also conducted to study the agreement between the two measurements.

## 3. Results

The evaluation of the angle phantom showed that the probe could reliably work along all tested orientations. For the three angles with designed values of 60, 40, and 20 degrees, the measured angles were 59.7 ± 2.9, 40.8 ± 1.9, and 20.9 ± 2.1 degrees, respectively, showing that the angles obtained by the probe closely agreed with the ground-truth values. In comparison, the measured angles for the conventional Scolioscan system were 60.1 ± 1.8, 41.2 ± 2.0, and 21.0 ± 1.9, respectively.

**Figure 6:**
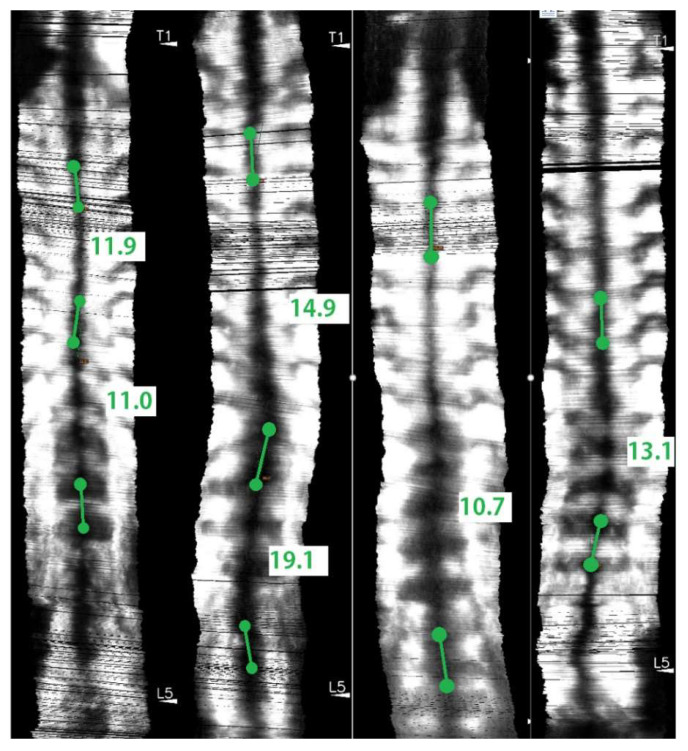
Coronal ultrasound images of subjects with various scoliosis severities using Scolioscan Air with SPA measurement.

For the 19 scoliosis patients scanned, there were six male and 13 female subjects, aged between seven and 24 years, and with the mean age of 13.6 ± 3.2 years. The 19 patients were scanned by both Scolioscan Air and conventional Scolioscan. For each patient, he/she was scanned once by both devices and one coronal image was obtained for each scan with similar image processing in both devices. Combining the thoracic curvatures and lumbar curvatures of 19 patients involved, total of 29 pairs of angles for the single visit were included for the analysis. Their spine curvatures measured by the Scolioscan system ranged from 6.4 to 33.0 degrees, with the mean of 15.1 ± 6.5 degrees. The mean value of SPA obtained by the portable 3D ultrasound imaging system was 16.4 ± 6.6 degrees (Figure 6).

**Figure 7:**
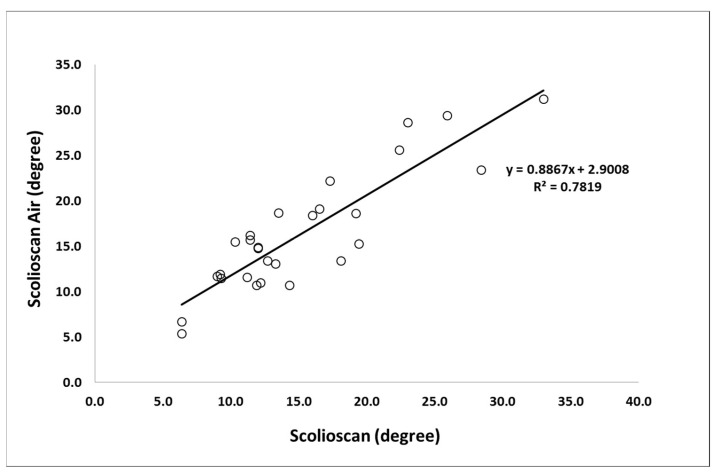
Correlation and equation between the SPA measurement results on Scolioscan Air and Scolioscan.

The high R^2^ value of 0.78 demonstrated a good correlation between the SPA results by the two systems (Figure 7).

**Figure 8:**
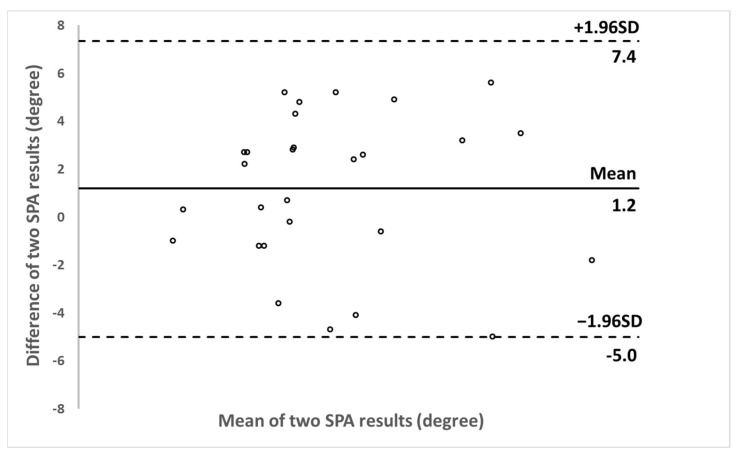
Bland-Altman plot between SPA results obtained by Scolioscan Air and Scolioscan.

The mean absolute difference between the SPA obtained by the two 3D ultrasound systems was 2.9 ± 1.8 degrees, with the maximum value of 5.6 degrees for one case. The Bland-Altman plot showed a small mean difference of 1.2 degrees and the differences were symmetrically distributed around the mean difference, and overall the two sets of SPA values agreed very well (Figure 8).

## 4. Discussion

This study reported the first validation study of a portable 3D ultrasound imaging system for scoliosis assessment. The results demonstrated that the portable version could achieve satisfactory coronal images for the curvature measurement and comparable scoliotic measurement results in comparison with the big Scolioscan machine which has already been in the market and widely used and evaluated [53,54,55]. The angle phantom test results also assured the accuracy of the angle measurement using this new probe. The conventional Scolioscan used a desktop ultrasound scanner for collecting B-mode ultrasound images and a big ferromagnetic spatial sensing system for 3D spatial information during scanning, while the newly developed portable 3D ultrasound imaging system adopted a palm-sized ultrasound module and a finger-sized optical 3D tracking sensor. There was a huge reduction of the dimension, which made the portable system (probe size of 182 × 92 × 150 mm and net weight of 450 g) be able to conduct scanning for subjects almost anywhere. The portable 3D ultrasound imaging system may have the potential to be applied in the whole management process of scoliosis, starting from screening, diagnosis, progression monitoring, treatment outcome assessment, as well as real-time feedback for optimizing conservative treatment, such as scoliosis-specific exercises.

Several previous studies have reported validation tests of the Scolioscan system for coronal curvature assessment, using X-ray Cobb angle measurement as a gold standard, and good correlations between the two methods have been demonstrated with R^2^ values of 0.78 [47], 0.86 [48], 0.76 [53], 0.94 [54], 0.76 [55], and 0.85 [68], respectively. In the present study, which is the first validation test for this newly developed portable 3D ultrasound imaging system to verify whether it could provide satisfied coronal images for scoliosis assessment, we did not use X-ray images as its reference for comparison. In the earlier validation study of the Scolioscan system, we noted that the ultrasound SPA measurement and X-ray Cobb angle measurements used different bony features for the curvature measurement, with the ultrasound relying on spinous process profile and X-ray on vertebral body plates. If we compare the results of the portable 3D ultrasound imaging system with those of X-ray images, it would be difficult to conclude whether the difference between the two was due to the different anatomical landmarks used between X-ray and ultrasound measurement. With this in mind, this study selected to first make a comparison between the portable 3D ultrasound imaging system with the Scolioscan to demonstrate that the two ultrasound systems can achieve comparable results. This study demonstrated that the R^2^ value between the scoliotic curvatures measured by the portable 3D ultrasound imaging system Scolioscan Air and the Scolioscan system was high as 0.78. Therefore, we could reasonably predict that the portable 3D ultrasound imaging system, Scolioscan Air, should be able to achieve similar results for scoliosis assessment, as the commercially available Scolioscan system. With such encouraging preliminary results about the performance of this portable Scolioscan, further studies with larger subject size and with X-ray Cobb angle as reference can be put in place to demonstrate its clinical potential for scoliosis management.

Despite the encouraging results, there are still several precautions to be taken in using Scolioscan Air, which are worthwhile for discussion. Most limitations of Scolioscan also exist in Scolioscan Air, as they share the same basic principle for free-hand 3D ultrasound imaging. Ultrasound image quality is subjected to scanning quality and some conditions of patients. Winged scapula or protruded scapula may obstruct the probe from scanning upwards, thus the probe needs to be titled slightly at those locations. A narrower probe with fan scanning may be a future solution. Poor contact or lack of gel between the probe and skin may include a dark region in the image, as ultrasound cannot be coupled into the tissues properly. Undesired movements of subjects may lead to blurry ultrasound images or distortion in the coronal images formed. Thick fat tissue layer along the spinal of the patient may induce attenuation to the ultrasound signals and affect the image quality. Therefore, patients with BMI larger than 25.0 kg/m^2^ were excluded from this study. This can be solved by using lower ultrasound frequency to enhance penetration, though the image resolution will be affected. Also, clear instructions to subjects and comprehensive training to operators are also recommended to ensure a good use of the device. At this moment, Scolioscan Air only provides the coronal view of spine. However, the ultrasound data set collected are in 3D, thus they can be analysed using 3D image software, which can provide sagittal [56] as well as the transverse view. Further studies of extracting transverse rotation using the transverse view images formed by the 3D software for the data collected by Scolioscan and Scolioscan Air are ongoing. In addition, the 3D image software can also provide the paraspinal muscles in 3D, which will be reported in our future papers.

We noted that the ultrasound image quality of the portable system was in general not as good as the Scolioscan system, though not affecting to show the overall spinal profile the curvature measurement. One potential reason was that the transmitting ultrasound power was limited for the palm-sized ultrasound module used in the system, due to the heating control. This led to the relatively shallow penetration in Scolioscan Air, even though both systems used 7.5 MHz linear array ultrasound transducer. This can be overcome by using low-power consumption chips thus reducing heating and increase transmitting power [69] or using an ultrasound transducer array with higher piezoelectric efficiency to achieve deeper penetration but with the same power consumption level [70].

The portable 3D ultrasound imaging system did not include any supporting device to stabilize the subject during scanning, and this may potentially induce motion artifacts in the images obtained. To support themselves during scanning, the subjects were asked to use their hands put on the surface of a vertical wall, and this is also a typical posture widely used for EOS scanning [71], its stability has been assured for most subjects. For X-ray or EOS, the scanning time is very short, while around 30 s was taken for ultrasound scanning and subject breath was not controlled during this period. We observed that most of the subjects could stabilize themselves steadily during the scanning, but some of them, particularly those youngsters, might move slightly. The movements of the subjects, particularly the lateral motions, could induce distortions in the coronal images generated, leading to the inaccurate measurement of spinal curvature. In this study, to achieve a fair comparison, the subjects used the same posture (without any external support) during the scanning for both systems. The Scolioscan has posture stabilization supporters provided but not used in this study [53]. If a subject was vulnerable to movement during scanning, he/she could move differently during the scanning with the portable system and during the Scolioscan scanning. This effect could double the motion artifacts caused in the difference between the measurement results provided by the two systems. In future studies and clinical applications, the motion of subjects during the scanning should be controlled as far as possible, with proper instruction to the subject. We also found some postures with the hands of subjects placed on the chest could also be good alternative postures. It is worthwhile doing further studies to evaluate the posture effect on the spine curvatures.

This new 3D ultrasound imaging system used a tracking camera to obtain 3D spatial information for spinal volume reconstruction and processing to form coronal view images. The tracking camera used 3D reference map for detecting its orientation and rotation data continuously during the movement, thus it first needed to build the reference map before scanning by moving the sensor along the intended tracking route. When the view of the camera was changed, the reference building procedure should be conducted again with green signal and smooth tracking path shown, and the system would give a notification for this to alert the operator. During the study, we found that it would be enough to conduct the reference building procedure once after the system was first turned on. If different subjects were always postured at a similar location, there was no further reference building needed unless the tracking has been distorted due to some reasons, such as moving objects included during scanning. One situation that the tracking could be distorted was the appearance of moving objects during scanning. This should be avoided no matter during the reference building procedure or 3D ultrasound scanning process. We noted that the tracking results could be affected by the interferences of moving objects, swinging cloths, moving body, within the view of the camera. At this stage, to guarantee a good use of the Scolioscan Air system in clinical applications, it is recommended to operate the device with proper training and make sure there are no moving objects within the view of the tracking camera. Further studies using multiple tracking cameras are being conducted, and the influences caused by moving objects can be compensated with more information acquired.

The sample size of this preliminary study for the validation of the newly developed novel portable system is relatively small, including six male and 13 female subjects. Their spinal curvature measured by the Scolioscan system ranged from 6.4 to 33.0 degrees, with the mean of 15.1 ± 6.5 degrees, thus most of these cases were mild to moderate scoliosis. With the feasibility of this portable 3D ultrasound imaging system demonstrated for scoliosis assessment, further validation studies can be followed up, involving more scoliosis patients and with more diverse scoliosis severities to demonstrate its clinical potentials.

Each imaging modalities have their own advantages and limitations. In comparison with Scolioscan, this new device has the advantage of being portable and can conduct scans for patients at almost any setting, clinic, community health center, school, or even home. This may greatly facilitate the early diagnosis and early treatment in the field of scoliosis management, as well as saving costs of examination and traveling for patients. In comparison with other radiation-free imaging systems, such as optical surface topography, one advantage of 3D ultrasound imaging is its ability to provide internal body structure of spine directly thus with high measurement accuracy for curvature measurement, and some limitations are its use of ultrasound gel and 30 s scanning time. The potential of paraspinal muscle assessment is another unique feature of 3D ultrasound imaging for spine in comparison with surface tomography or X-ray imaging. Therefore, users can select a suitable imaging modality for their specific needs of patients or use a number of modalities in a complementary matter. Looking forward, when this newly developed portable 3D ultrasound imaging device can be widely adopted in the field of scoliosis care, it may also generate positive economic impacts. We can move this 3D imaging device to the scoliosis patients, such as in clinics, community centers, and even homes, when Scolioscan Air is widely installed. This will save a lot of traveling time and costs. The machine as well as the operating costs of Scolioscan Air will be just a very small portion of other imaging devices, such as X-ray, and thus the economic burden to the patients and the overall healthcare system can be reduced. Scolioscan Air may provide an accurate while convenient tool for the mass screen, early diagnosis and treatment, as well as frequent progression monitoring. This may potentially help to reduce the number of severe cases of scoliosis, thus potentially avoiding very invasive and expensive surgical procedures. Martin et al. (2012) reported that the mean hospital charges for AIS spinal fusions increased from $72,780 in 2001 to $155,278 in 2011 (113% increase), averaging 11.3% annually (*p* < 0.0001) [72]. According to National Scoliosis Foundation, there are annually around 38,000 patients undergo spinal fusion surgery [73]. Therefore, the positive economic values of this newly developed device would be even higher if we count the number of severe cases that it can help to avoid.

## 5. Conclusions

A portable 3D ultrasound imaging system, namely Scolioscan Air, was successfully developed for the radiation-free assessment of scoliosis, with much less venue restriction in comparison with its earlier version, Scolioscan. The SPA obtained by Scolioscan Air agreed very well with those obtained by Scolioscan, demonstrating the measurement of the two systems were comparable. Scolioscan Air may have great potentials to be a reliable measurement modality for quantitative evaluation of spinal deformity in scoliosis. With its portable feature, it can be widely used for scoliosis screening, diagnosis, curve progression monitoring, treatment outcome assessment, and scientific research. Further studies with a larger number of subjects with diverse scoliotic curves are suggested to fully demonstrate the potentials of this novel portable 3D ultrasound imaging system.

## 6. Patents

This work is related to a number of patents for which Y.-P.Z. is an inventor, including CN201080040696, US8900146B2, EP2459073B1, and CN201210163528.

## Data Availability

Not applicable.
